# A new species of *Hisonotus* (Siluriformes, Loricariidae) from rio São Francisco basin, Brazil

**DOI:** 10.3897/zookeys.498.6896

**Published:** 2015-04-21

**Authors:** Fábio F. Roxo, Gabriel S. C. Silva, Claudio Oliveira

**Affiliations:** 1 Universidade Estadual Paulista, Departamento de Morfologia, Laboratório de Biologia e Genética de Peixes, Rubião Júnior s/n, 18618970 Botucatu, São Paulo State, Brazil

**Keywords:** Cascudinhos, Fresh-Water, Minas Gerais State, Neotropical Fish, Otothyrinae

## Abstract

A new species of *Hisonotus* is described from the rio São Francisco basin. The new species can be distinguished from congeners by having (1) a unique coloration pattern of caudal fin with one black spot extending from its origin to the ventral lobe and two dark spots at the end of the lobe’s rays; (2) odontodes forming longitudinally aligned rows on head and trunk; (3) a functional *V*-shaped spinelet; (4) a single rostral plate at the tip of the snout; (5) by lacking contrasting dark geometric spots on the anterodorsal region of the body; (6) a lower caudal-peduncle depth; and (7) lower counts of the lateral median plates and (8) higher premaxillary and dentary teeth. The new species is the second described species of the genus *Hisonotus* in the rio São Francisco basin. It was found inhabiting the marginal vegetation of the rio São Francisco and three of its tributary, rio das Velhas, rio Paraopeba and rio Formoso.

## Introduction

Loricariidae is one of the largest and most diverse families of Neotropical freshwater fish with about 900 valid species (Eschmeyer and Fong 2014). The loricariids are subdivided into seven subfamilies: Delturinae, Hypoptopomatinae, Hypostominae, Lithogeninae, Loricariinae, Neoplecostominae, and Otothyrinae (Armbruster 2004; Reis et al. 2006; Chiachio et al. 2008; Roxo et al. 2014a). Currently, Otothyrinae (sensu Chiachio et al. 2008 and Roxo et al. 2014a) is composed of about 100 valid species classified in twelve genera (Eschmeyer 2014).

Within the Otothyrinae, the genus *Hisonotus* Eigenmann & Eigenmann, 1889 (type species *Hisonotus notatus* Eigenmann & Eigenmann, 1889) was resurrected by Schaefer (1998) with the following combination of characters: snout plates in the anterior portion of the nostril reduced or absent, the rostrum having enlarged odontodes, and the lateral rostral margin composed of thickened plates. However, Britski and Garavello (2007) considered the rostrum with enlarged odontodes as a character very polymorphic and present in several other genera and species of Otothyrinae, as in species of *Parotocinclus* Eigenmann & Eigenmann, 1889. Furthermore, Britski and Garavello (2007) suggested that the other two characters are not satisfactory to define the genus *Hisonotus*.

Currently, *Hisonotus* contains 34 valid species, 19 of which were described in the past decade (Eschmeyer 2014), representing an increase of 127% of the diversity of this genus. Herein, we add a new species to the genus *Hisonotus* found during recent collection expeditions to the rio São Francisco basin. This is the second species of the genus described from this hydrographic system.

## Material and methods

Measurements and counts were taken from the left side of the fish, and were made from point to point to the nearest 0.1 mm with a digital caliper. Abbreviations used in the text and the measurements followed Carvalho and Reis (2009). Specimens were cleared and double stained (c&s) according to the method of Taylor and Van Dyke (1985). Vertebral counts also include the five vertebrae that comprise the Weberian apparatus and the compound caudal centrum (PU1 + U1) as one element. Dorsal-fin ray counts include the spinelet as the first unbranched ray. Institutional acronyms follow Fricke and Eschmeyer (2014). Specimens are deposited at the LBP, Laboratório de Biologia e Genética de Peixes, Universidade Estadual Paulista, Botucatu; MZUSP, Museu de Zoologia, Universidade de São Paulo, São Paulo. Zoological nomenclature follows the International Code of Zoological Nomenclature (International Commission on Zoological Nomenclature 1999).

## Results

### Hisonotus vespuccii sp. n.

Taxon classificationAnimaliaSiluriformesLoricariidae

http://zoobank.org/CD9657F5-3F02-4B32-BF89-24AF86020201

**Figure 1.**
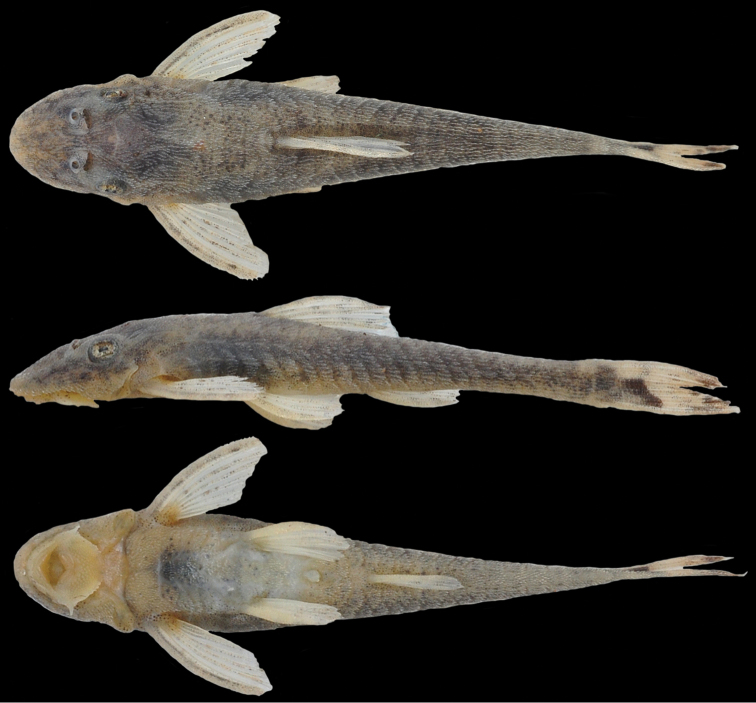
Holotype of *Hisonotus vespuccii*, MZUSP 115274, female, 32.6 mm SL, from Minas Gerais State, municipality of Pirapora, rio São Francisco, 17°21'00"S, 44°57'08"W.

Figure 1 , 3 ; Table 1

#### Holotype.

MZUSP 115274 (female 32.6 mm SL) Brazil, Minas Gerais State, municipality of Pirapora, rio São Francisco, 17°21'00"S, 44°57'08"W, 11 November 2014, LE Ochoa, FF Roxo, LH Roxo, GSC Silva.

#### Paratypes.

All from Brazil, Minas Gerais State, rio São Francisco basin (249 specimens in total). LBP 8960 (4 females 32.3–35.8 mm SL, 3 males 27.0–28.2 mm SL), municipality of Presidente Juscelino, rio das Velhas, 18°40'21"S, 44°11'33"W, 01 October 2009, C Oliveira, FF Roxo, GJC Silva, BF Melo. LBP 10421 (9 females 27.7–30.3 mm SL, 9 males 23.6–26.5 mm SL, 5 c&s sex not determined 20.2–29.6 mm SL), municipality of Pirapora, rio São Francisco, 17°21'00"S, 44°57'08"W, 15 July 2010, JA Senhorini, M Mehanna. LBP 19491 (9 females 19.9–32.4 mm SL, 12 males 21.1–27.9 mm SL, 1 female c&s 32.6 mm SL, 1 male c&s 27.7 mm SL), collected with holotype. LBP 19495 (1 female 32.6 mm SL, 1 male 28.7 mm SL), municipality of Presidente Juscelino, rio das Velhas, 18°40'21"S, 44°11'33"W, 11 November 2014, LE Ochoa, FF Roxo, LH Roxo, GSC Silva. MZUSP 39208 (5 sex not determined 23.8–30.0 mm SL), rio São Francisco, 29 November 1987, Y Sato (UHE Formoso project). MZUSP 39280, (3 sex not determined 28.4–30.2 mm SL), rio São Francisco, 20 January 1988, Y Sato (UHE Formoso project). MZUSP 39351 (33 sex not determined 16.5–34.4 mm SL), rio São Francisco, 23 January 1988, Y Sato (UHE Formoso project). MZUSP 39446 (9 sex not determined 22.9–30.5 mm SL), rio Formoso, 08 February 1988, Y Sato (UHE Formoso project). MZUSP 39482 (1 sex not determined 24.5 mm SL), rio Formoso, 08 to 10 February 1988, Y Sato (UHE Formoso project). MZUSP 39511 (1 sex not determined 30.4 mm SL), córrego Marambaia, rio São Francisco, 09 February 1988, Y Sato (UHE Formoso project). MZUSP 51507 (2 sex not determined 25.6–31.3 mm SL), municipality of Fortuna de Minas, rio Paraopeba, 13 July 1995, CBM Alves. MZUSP 57587 (110 sex not determined 20.8–33.6 mm SL), street between municipalities of Itacarambí and Manga, córrego das Missões, 15 July 1993, RE Reis. MZUSP 57588 (7 sex not determined 20.2–24.9 mm SL), street between municipalities of Manga and Montalvânia, rio Japuré, rio São Francisco, 15 July 1993, RE Reis. MZUSP 57590 (20 sex not determined 22.8–31.4 mm SL), municipality of Januária, rio São Francisco, 15°29'25"S, 44°21'50"W, 14 July 1993, JC Garavello. MZUSP 57591 (3 sex not determined 23.1–26.5 mm SL), street between municipalities of Manga and Montalvânia, rio Calindó, rio São Francisco, 15 July 1993, RE Reis.

#### Diagnosis.

*Hisonotus vespuccii* differs from the congeners by having a unique coloration pattern of caudal fin with one black spot extending from its origin to the ventral lobe and two dark spots at the end of the lobe`s rays and the following combination of character states (none is unique): odontodes forming longitudinally aligned rows (one odontode after the other, but not necessarily forming parallel series) on head and trunk; a functional *V*-shaped spinelet; the presence of a single rostral plate at tip of the snout; the lack of contrasting dark geometric spots on the anterodorsal region of the body; a low caudal peduncle (depth 6–8% SL); few lateral median plates (21–23); and numerous premaxillary and dentary teeth (13–21 and 11–21, respectively).

#### Description.

Counts and measurements are presented in Table 1. Maximum body size 35.7 mm SL. Dorsal profile of head, in lateral view, slightly convex from snout tip to margin of posterior naris; strongly convex to posterior margin of parieto-supraoccipital; and almost straight to dorsal-fin origin. Dorsal profile of trunk, in lateral view, straight and descending from dorsal-fin origin to insertion of caudal-fin. Ventral profile, in lateral view, straight from snout tip to anal-fin origin; concave and ascending to caudal-fin insertion. Greatest body depth at dorsal-fin origin (14−18% SL). Greatest body width at cleithral region (21−25% SL), progressively narrowing towards to both snout and caudal fin. Cross-section of body between pectoral and pelvic fins dorsally rounded and ventrally flat; cross-section of caudal peduncle ellipsoid, rounded laterally and almost flat dorsally and ventrally.

**Table 1.** Morphometrics and meristic data for *Hisonotus vespuccii*, holotype and paratypes measured are from LBP fish collection.

Character	Holotype	Males, n = 21			Females, n = 24			All paratypes, n = 45	
		Range	Mean	SD	Range	Mean	SD	Mean	SD
**SL**	**32.6**	**24.1−28.7**	**26.2**	**1.4**	**24.4−35.7**	**30.2**	**2.8**	**28.3**	**3.0**
**Percents of SL**									
Head length	33.1	34.1−37.6	36.1	0.8	32.7−38.1	35.6	1.3	35.8	1.1
Predorsal length	44.5	44.2−47.3	45.8	0.8	44.1−48.6	46.1	1.2	46.0	1.0
Dorsal-fin spine length	22.6	22.6−27.2	24.6	1.3	19.5−25.4	23.2	1.1	23.8	1.4
Anal-fin unbranched ray length	15.5	16.0−19.2	17.4	0.8	14.9−18.1	16.1	0.7	16.7	1.0
Pectoral-fin spine length	23.9	23.9−29.1	26.0	1.3	23.1−27.7	25.2	1.1	25.6	1.3
Pelvic-fin unbranched ray length	16.9	17.3−21.5	19.3	1.0	15.1−18.5	16.8	0.8	18.0	1.5
Cleithral width	21.5	22.3−24.3	23.2	0.5	21.2−24.9	23.0	0.8	23.1	0.6
Thoracic length	18.0	15.2−18.3	16.5	0.7	14.9−18.4	16.7	0.9	16.6	0.8
Abdominal length	22.6	20.7−23.1	21.6	0.5	20.1−24.6	22.5	1.1	22.1	1.0
Body depth at dorsal-fin origin	14.7	14.0−17.7	15.7	0.9	14.3−17.5	15.9	0.8	15.8	0.9
Caudal-peduncle length	39.0	36.7−40.3	38.6	0.8	34.0−39.4	37.3	1.2	37.9	1.2
Caudal-peduncle depth	6.2	6.9−8.0	7.4	0.3	6.2−7.9	7.0	0.4	7.2	0.4
Nares opening	12.5	12.9−18.0	15.3	1.3	9.6−12.5	11.1	0.7	13.0	2.3
**Percents of HL**									
Snout length	48.8	45.8−50.6	48.7	1.2	45.5−50.3	48.9	1.1	48.8	1.2
Orbital diameter	16.1	13.5−16.6	15.5	0.7	13.4−16.9	14.9	0.9	15.2	0.9
Interorbital width	36.1	35.1−38.7	37.1	0.9	34.1−39.0	36.2	1.3	36.6	1.2
Head depth	40.5	39.0−45.0	42.0	1.5	38.6−47.2	42.5	1.9	42.3	1.7
Suborbital depth	17.2	14.9−18.8	16.6	0.9	14.9−20.3	17.0	1.6	16.8	1.3
Mandibular ramus	9.7	8.1−10.5	9.3	0.6	8.3−15.6	9.6	1.4	9.5	1.1
**Meristics**									
Left lateral scutes	22	21−23	22	-	21−23	22	-	22	-
Left premaxillary teeth	19	15−21	17	-	13−21	19	-	17	-
Left dentary teeth	19	11−19	14	-	13−21	16	-	16	-

Head rounded in dorsal view; snout round and slightly pointed. Dorsal and ventral series of odontodes along anterior margin of snout completely covering its tip; odontodes larger than remaining ones on head. Odontodes on head and trunk hypertrophied and arranged in longitudinal rows. Head without conspicuous crests. Some specimens with a poor developed tuft of odontodes in posterior portion of parieto-supraoccipital. Eyes small (13–17% HL), dorsolaterally positioned. Iris operculum present and developed. Premaxillary teeth 13–21; dentary teeth 11–21. Teeth bifid, major (medial) cusp large and rounded, minor (lateral) cusp minute and pointed. Accessory patch of teeth absent on dentary and premaxilla. Oral disk oval, covered with papillae uniformly distributed on base of dentary and premaxilla and slightly decreasing in size distally. Lower lip larger than upper lip; its border fringed. Maxillary barbel present and joined to lower lip. Presence of conspicuous *V*-shaped buccal papilla located immediately anterior to buccal valve. Tip of snout with large rostral plate.

Dorsal fin II,7; its origin slightly posterior to pelvic-fin origin. Tip of adpressed dorsal fin surpassing vertically through end of anal-fin origin. Dorsal-fin spinelet short and *V*-shaped; dorsal-fin lock functional. Pectoral fin I,6; its tip reaching middle of pelvic-fin length when depressed. Pectoral-axillary slit present between pectoral-fin insertion and lateral process of cleithrum. Pectoral spine supporting sharp odontodes on dorsal and ventral surfaces (well developed posteriorly). Pelvic fin I,5; its tip reaching anal-fin origin when depressed in males and far from reaching anal-fin origin in females. Pelvic-fin unbranched ray with dermal flap along its dorsal surface in males. Pectoral spine supporting sharp odontodes on ventral surface turned mesially.

Anal fin i,5; its tip reaching seventh or eighth plate from its origin. Caudal-fin i,7–7,I; distal margin forked. Adipose fin absent. Total vertebrae 27 (in 7 c&s specimens). Body almost entirely covered by bone plates, except on ventral portion of head, around pectoral- and pelvic-fin origins, on dorsal-fin base and area around anus. Abdomen partially covered by bony plates randomly distributed and surrounded by naked areas (in some specimens abdomen is completely covered by bony plates). Laterally, body completely covered by plates; mid-dorsal and mid-ventral plate series well developed reaching vertical through half of caudal peduncle; median plate series continuous in median portion of body. Coracoid and cleithrum completely exposed, covered with odontodes. Arrector fossae partially enclosed by ventral lamina of coracoids.

#### Color in alcohol.

**Figure 2.**
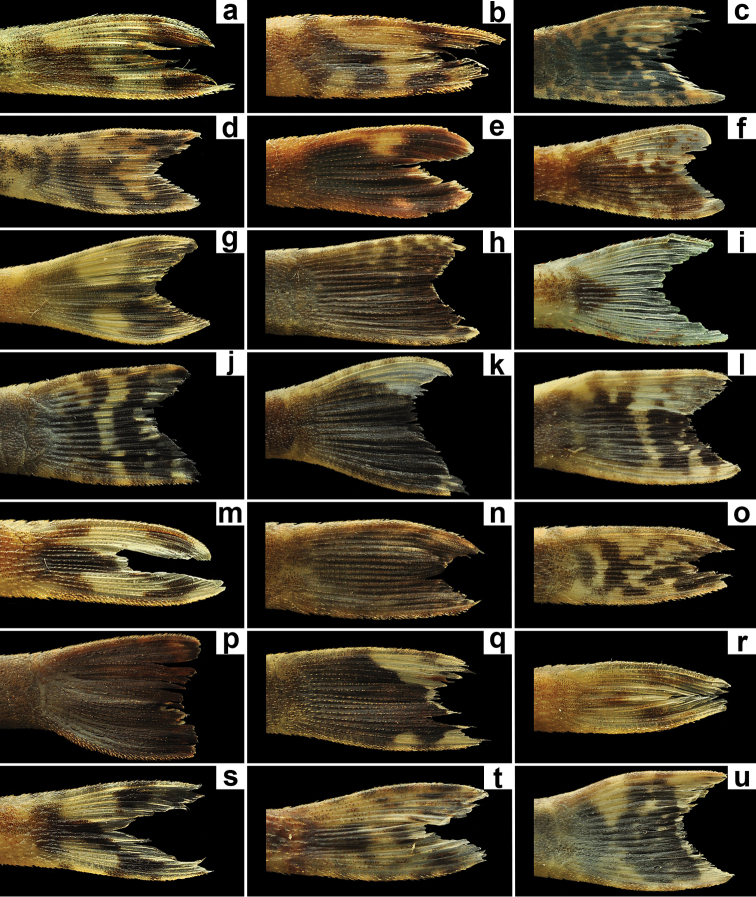
Coloration pattern of caudal fin of *Hisonotus* species. **a** *Hisonotus vespuccii*, LBP 19491, 26.2 mm SL **b** *Hisonotus acuen*, LBP 16279, 26.1 mm SL **c** *Hisonotus armatus*, LBP 14461, 38.2 mm SL **d** *Hisonotus bocaiuva*, LBP 17402, 24.8 mm SL **e** *Hisonotus chromodontus*, LBP 7964, 27.6 mm SL **f** *Hisonotus depressicauda*, LBP 1293, 26.1 mm SL **g** *Hisonotus francirochai*, LBP 8356, 31.6 mm SL **h** *Hisonotus heterogaster*, LBP 14580, 38.7 mm SL **i** *Hisonotus insperatus*, LBP 17432, 26.9 mm SL **j** *Hisonotus iota*, LBP 13072, 41.5 mm SL **k** *Hisonotus leucofrenatus*, LBP 2039, 40.7 mm SL **l** *Hisonotus leucophrys*, LBP 13071, 41.1 mm SL **m** *Hisonotus luteofrenatus*, LBP 19534, 30.5 mm SL **n** *Hisonotus megaloplax*, LBP 13108, 35.8 mm SL **o** *Hisonotus montanus*, LBP 13055, 31.8 mm SL **p** *Hisonotus nigricauda*, LBP 14652, 38.5 mm SL **q** *Hisonotus notatus*, LBP 18472, 32.0 mm SL **r** *Hisonotus oliveirai*, LBP 13333, 24.0 mm SL **s** *Hisonotus paresi*, LBP 13351, 24.6 mm SL **t** *Hisonotus piracanjuba*, LBP 17256, 22.1 mm SL **u** *Hisonotus vireo*, LBP 14452, 35.5 mm SL.

Ground color of dorsal surface of head and body dark gray to lighter brown (juveniles lighter than adults). Ventral surface light brown to yellow in juveniles. All body and fins covered by scattered chromatophores, more visible on ventral portions and around fins insertions (Fig. 1). Caudal-fin hyaline, except for one black spot at its origin extending to ventral lobe and two dark spots at end of rays (Fig. 2a). In some specimens, caudal-fin with chromatophores irregular distributed and sometimes badly forming two dark strips (more visible in juveniles). Neither variation nor variability of caudal-fin coloration patter found in samples we examined (holotype and 249 specimens widely distributed in rio São Francisco basin) with specific emphasis to variability between populations and variation depending on feeding.

#### Color in life.

**Figure 3.**
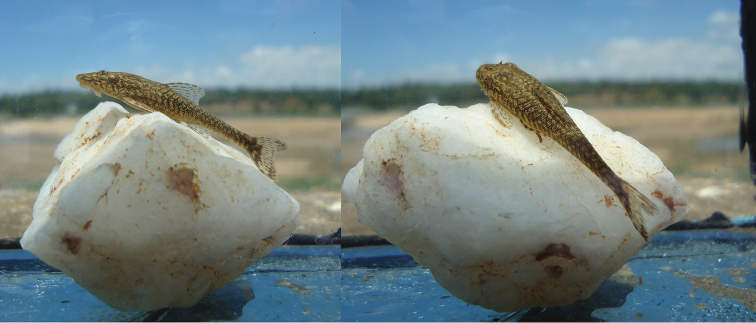
Live specimen of *Hisonotus vespuccii*, LBP 19491, paratype, 32.4 mm SL, from Minas Gerais State, municipality of Pirapora, rio São Francisco.

Similar to pattern described for alcohol individuals, but with ground color light green (Fig. 3).

#### Sexual dimorphism.

Adult males distinguished from females by five characters: (1) presence of a papilla at urogenital opening in males (*vs.* papilla absent in females); (2) pelvic-fin extending beyond anal-fin origin in males, mean 19% SL (*vs.* pelvic fin far from reaching anal-fin origin in females, mean 17% SL); (3) unbranched pelvic-fin ray supporting a dermal flap (flap slightly wider in basal portion and progressively narrowing distally) on proximal dorsal surface in males (*vs.* dermal flap absent in females); (4) nares opening wider in males (13–18% HL) than females (10–13% HL); (5) body size smaller in males (mean 26 mm SL) and larger in females (mean 30 mm SL). See Table 1 for values of morphometric characters between males and females.

#### Habitat and distribution.

**Figure 4.**
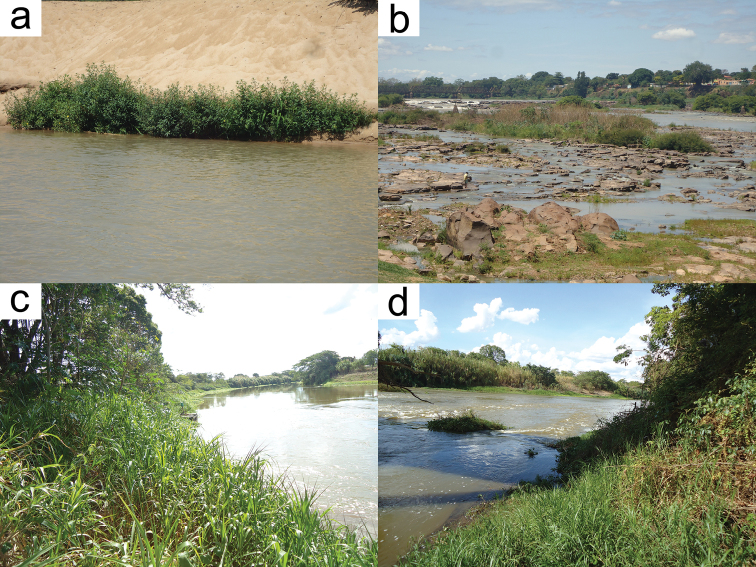
(a) (b) Type locality of *Hisonotus vespuccii* at rio São Francisco, municipality of Pirapora, Minas Gerais State, 17°21'00"S, 44°57'08"W. (c) (d) Paratypes locality of *Hisonotus vespuccii* at rio das Velhas, municipality of Presidente Juscelino, Minas Gerais State, 18°40'21"S, 44°11'33"W. Photo: LH Roxo.

**Figure 5.**
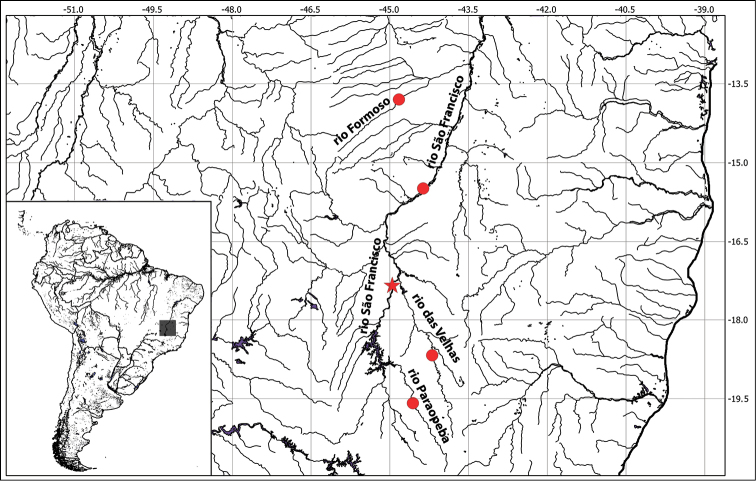
Map of the distribution of *Hisonotus vespuccii*. Red Star = type locality, at rio São Francisco, municipality of Pirapora, Minas Gerais State. Red Circles = paratypes localities of *Hisonotus vespuccii* at rio das Velhas, rio Paraopeba and rio Formoso, Minas Gerais State.

*Hisonotus vespuccii* was found associated with marginal vegetation (Fig. 4) in the rio São Francisco and in three of its tributaries, rio das Velhas, rio Paraopeba and rio Formoso (Fig. 5). The new species seems to be abundant through all rio São Francisco basin.

#### Etymology.

The specific name “vespuccii” comes from Italian and is in reference to Américo Vespúcio (Amerigo Vespucci in Italian), navigator and explorer to whom is attributed the discovery of the rio São Francisco in 1501.

## Comparative remarks and discussion

The new species *Hisonotus vespuccii* has one character proposed by Schaefer (1998) to diagnose the genus *Hisonotus*: the rostrum with enlarged odontodes. Moreover, the new species also shares three characters with many species of *Hisonotus*: a single rostral plate on the tip of the snout, an arrector fossae partially enclosed by a ventral lamina of the coracoid, a character also used by Schaefer (1998) as synapomorphy of all Otothyrini except the New Taxon 3, and a functional *V*-shaped spinelet. This last character was firstly proposed by Carvalho and Datovo (2012) with pers. comm. of Roberto E. Reis, and posteriorly was reported by Silva et al. (2014) as a possible synapomorphy that may help delimit a new genus within *Hisonotus*. However, to better understand the relationship of the new species with other species assigned to *Hisonotus* a phylogenetic analysis is still necessary.

We used seven characters to distinguish the new species *Hisonotus vespuccii* from congeners. The first character was the caudal fin with one black spot extending from its origin to the ventral lobe and two dark spots at the end of the lobe’s rays, a pattern unique among *Hisonotus* species (see Fig. 2b–u for caudal-fin coloration pattern of some species of *Hisonotus*). Britski and Garavello (2007) discussing about the coloration pattern of the teeth in *Hisonotus chromodontus* Britski & Garavello, 2007 suggested that this aspect of the organism could be a result of physiological features changing according to the individual’s foraging success and physiological efficiency, as well as according to the characteristics of the water where the species lives. However, the pattern of caudal-fin coloration seems to be conserved among species of *Hisonotus* (Fig. 2), with a pattern varying more drastically in *Hisonotus acuen* Silva, Roxo & Oliveira, 2014, a species widely distributed through headwaters of the rio Xingu basin (see Fig. 5 in Silva et al. 2014). In *Hisonotus vespuccii*, the pattern of the caudal-fin with one black spot extending from its origin to the ventral lobe and two dark spots at the end of the lobe`s rays is present in the holotype and the 249 paratypes analyzed.

Moreover, the new species *Hisonotus vespuccii* was distinguished from congeners by present a combination of the following characters: (2) odontodes forming longitudinally aligned rows (one odontode after the other, but not necessarily forming parallel series) on head and trunk, a character shared with *Hisonotus insperatus*, *Hisonotus luteofrenatus*, *Hisonotus oliveirai*, and *Hisonotus paresi*; (3) a functional *V*-shaped spinelet as reported previously (see Fig. 2 in Silva et al. 2014 to description of this character state) shared with *Hisonotus acuen*, *Hisonotus bockmanni*, *Hisonotus chromodontus*, *Hisonotus insperatus*, *Hisonotus luteofrenatus*, *Hisonotus oliveirai*, *Hisonotus paresi*, and *Hisonotus piracanjuba*; (4) the presence of a single rostral plate at tip of the snout, a character also present in *Hisonotus insperatus*, *Hisonotus luteofrenatus*, *Hisonotus oliveirai*, *Hisonotus paresi*, and *Hisonotus piracanjuba*; (5) the lacking of contrasting dark geometric spots on the anterodorsal region of the body, a character used to distinguish the new species of *Hisonotus bockmanni* and *Hisonotus paresi*; (6) a lower caudal-peduncle depth (6.2–8.0% of SL) used to distinguish the new species of *Hisonotus acuen* (8.6–11.1% of SL); and (7) lower counts of the lateral median plates (21–23) and higher counts of premaxillary (13–21) and dentary teeth (11–21) used to distinguish *Hisonotus vespuccii* of *Hisonotus insperatus* (25–26; 10–12; 8–11, respectively).

Members of Loricariidae are known to have intense sexual dimorphisms (Py-Daniel and Fernandes 2005) as we can observe in species of the genera *Ancistrus* Kner, 1854 (Sabaj et al. 1999), *Neoplecostomus* Eigenmann & Eigenmann, 1888 (Zawadzki et al. 2008; Roxo et al. 2012; Andrade and Langeani 2014), *Pareiorhaphis* Miranda Ribeiro, 1918a (Pereira et al. 2007), *Hisonotus* (Martins and Langeani 2012), *Hypostomus* Lacepède, 1803 and *Chaetostoma* Tschudi, 1846 (Nomura and Mueller 1980; Lopez and Roman-Valencia 1996), *Farlowella* Eigenmann & Eigenmann, 1889 (Retzer and Page 1996) and many other. In *Hisonotus vespuccii*, we observed five sexual dimorphic characters: the presence of a papilla at the urogenital opening, a pelvic-fin that extends beyond the anal-fin origin, the unbranched pelvic-fin ray supporting a dermal flap on their proximal dorsal surface, the nares opening wider and a body size that seems to be smaller in males than in females. The first three characters are very common among species of *Hisonotus*, however differences in size of nares were only previously reported in the original description of *Hisonotus piracanjuba* (Martins and Langeani 2012) and differences in body size in *Hisonotus ringueleti* (Aquino et al. 2001). Carvalho and Reis (2009) reported that the presence of the dermal flap on proximal dorsal surface of pelvic-fin on males is a plesiomorphic character shared among most members of Otothyrinae and that the derived condition evolved several times within this subfamily at the genera *Schizolecis* Britski & Garavello, 1984, *Epactionotus* Reis & Schaefer, 1998, and within Hypoptopomatinae in *Acestridium* Haseman, 1911, *Oxyropsis* Eigenmann & Eigenmann, 1889 and *Hypoptopoma* Günther, 1868.

### Comparative material

All from Brazil, except when stated otherwise.

*Hisonotus acuen* Silva, Roxo & Oliveira, 2014: MZUSP 115350, 1, 25.9 mm SL, holotype, tributary of rio Toguro, Querência, Mato Grosso State; LBP 15755, 16, 19.5–26.0 mm SL, paratypes, tributary of rio Suiá-Missu, ribeirão Cascalheira, Mato Grosso State; LBP 16274, 27, 20.2–29.1 mm SL, 2 c&s, 23.6−24.2 mm SL, paratypes, tributary of rio Culuene, Gaúcha do Norte, Mato Grosso State; LBP 16275, 29, 16.7–25.2 mm SL, 2 c&s, 19.3−20.8 mm SL, paratypes, tributary of rio Feio, Querência, Mato Grosso State; LBP 16278, 12, 18.8–25.1 mm SL, 2 c&s, 26.8−27.1 mm SL, paratypes, córrego Xavante, Primavera do Leste, Mato Grosso State.

*Hisonotus aky* (Azpelicueta, Casciotta, Almirón & Koerber, 2004): MHNG 2643.039, 2, 33.1−34.2 mm SL, paratypes, arroio Fortaleza, Argentina.

*Hisonotus armatus* Carvalho, Lehmann, Pereira & Reis, 2008: MZUSP 93884, 5, 37.6–44.4 mm SL, paratypes, arroio Arambaré, Pedro Osório, Rio Grande do Sul State.

*Hisonotus bocaiuva* Roxo, Silva, Oliveira & Zawadzki, 2013: MZUSP 112204, 1, 24.2 mm SL, holotype, córrego Cachoeira, Bocaiúva, Minas Gerais State; LBP 9817, 9, 3 c&s, 18.3−23.2 mm SL, paratypes, córrego Cachoeira, Bocaiúva, Minas Gerais State.

*Hisonotus brunneus* Carvalho & Reis, 2011: MZUSP 104947, 4, 37.2−41.3 mm SL, paratypes, rio Passo Novo, Cruz Alta, Rio Grande do Sul State.

*Hisonotus carreiro* Carvalho & Reis, 2011: MCP 40943, 3, 33.6−35.8 mm SL, arroio Guabiju, Guabiju, Rio Grande do Sul State.

*Hisonotus charrua* Almirón, Azpelicueta, Casciotta & Litz, 2006: LBP 4861, 1, 35.9 mm SL, arroio Guaviyú, Artigas, Uruguay; MHNG 2650.051, 1, 34.2 mm SL, paratype, arroio Aspinillar, Uruguay.

*Hisonotus chromodontus* Britski & Garavello, 2007: LBP 7964, 25, 24.0−28.3 mm SL, 4 c&s, 24.9−28.9 mm SL, rio dos Patos, Nova Mutum, Mato Grosso State; LBP 7974, 26, 17.7–24.8 mm SL, rio dos Patos, Nova Mutum, Mato Grosso State; LBP 12278, 2, 26.7−28.7 mm SL, 1 c&s, 26.7 mm SL, rio Sumidouro, Tangará da Serra, Mato Grosso State; MZUSP 45355, 1, 25.9 mm SL, holotype, tributary of rio Preto, Diamantino, Mato Grosso State; MZUSP 70758, 7, 19.4−23.9 mm SL, paratype, riacho Loanda, Sinop, Mato Grosso State; NUP 10924, 24, 19.5−31.5 mm SL, rio Preto, Diamantino, Minas Gerais State.

*Hisonotus depressicauda* (Miranda Ribeiro, 1918b): MZUSP 5383, 1, 24.4 mm SL, paralectotype, Sorocaba, São Paulo State; LBP 17474, 5 c&s, 18.1−24.0 mm SL, rio Araquá, Botucatu, São Paulo State.

*Hisonotus francirochai* (Ihering, 1928): LBP 13923, 22, 25.7−35.7 mm SL, córrego sem nome, Capetinga, Minas Gerais State; MZUSP 3258, 1, 29.4 mm SL, lectotype, rio Grande, São Paulo State.

*Hisonotus heterogaster* Carvalho & Reis, 2011: LBP 3335, 39, 20.8−30.1 mm SL, arroio sem nome, rio Grande, Rio Grande do Sul State; MZUSP 104948, 3, 40.3−43.0 mm SL, paratypes, arroio Felício, Júlio de Castilho, Rio Grande do Sul State.

*Hisonotus insperatus* Britski & Garavello, 2003: LBP 4945, 5, 27.3−28.5 mm SL, 2 c&s, 28.2−29.9 mm SL, Botucatu, São Paulo State; LBP 6770, 5, 25.1−28.2 mm SL, 3 c&s, 20.0−27.0 mm SL, ribeirão Cubatão, Marapoama, São Paulo State; LBP 13336, 1 c&s, 26.0 mm SL, rio Capivara, Botucatu, São Paulo State; LBP 13337, 2 c&s, 27.4−28.6 mm SL, rio Araquá, Botucatu, São Paulo State; MZUSP 22826, 1, 25.4 mm SL, paratype, córrego Água Tirada, Três Lagoas, Minas Gerais State; MZUSP 24832, 1, 23.8 mm SL, paratype, rio Corumbataí, Corumbataí, São Paulo State; MZUSP 78957, 1, 29.6 mm SL, holotype, rio Capivara, Botucatu, São Paulo State; MZUSP 78960, 31, 12.6−26.0 mm SL, paratypes, 5 c&s, 22.7−24.7 mm SL, rio Pardo, Botucatu, São Paulo State; MZUSP 78965, 10, 15.6−28.6 mm SL, paratypes, rio Araquá, Botucatu, São Paulo State; MZUSP 78968, 5, 24.1−27.3 mm SL, paratypes, córrego da Figueira, Lins, São Paulo State.

*Hisonotus iota* Carvalho & Reis, 2009: LBP 13072, 5, 32.3−33.0 mm SL, rio Chapecó, Coronel Freitas, Santa Catarina State.

*Hisonotus laevior* Cope, 1894: LBP 3377, 1, 25.2 mm SL, arroio dos Corrientes, Pelotas, Rio Grande do Sul State; LBP 6037, 8, 33.4−47.0 mm SL, rio Maquiné, Osório, Rio Grande do Sul State; LBP 13187, 7, 19.4−45.8 mm SL, córrego sem nome, Camaquá, Rio Grande do Sul State.

*Hisonotus leucofrenatus* (Miranda Ribeiro, 1908): LBP 2085, 7, 38.3−50.6 mm SL, rio Sagrado, Morretes, Paraná State; LBP 6837, 36, 35.1−43.5 mm SL, rio Fau, Miracatu, São Paulo State.

*Hisonotus leucophrys* Carvalho & Reis, 2009: LBP 13065, 6, 17.2−33.6 mm SL, rio Ariranhas, Xavantina, Santa Catarina State; LBP 13073, 1, 36.8 mm SL, rio Guarita, Palmitinho, Rio Grande do Sul State.

*Hisonotus luteofrenatus* Britski & Garavello, 2007: MZUSP 62593, 1, 28.6 mm SL, holotype, córrego Loanda, Cláudia, Mato Grosso State; MZUSP 62594, 8, 22.4−30.5 mm SL, paratypes, riacho Selma, Sinop, Mato Grosso State; MZUSP 87144, 8, 16.8−27.9 mm SL, paratypes, córrego Loanda, Cláudia, Mato Grosso State.

*Hisonotus megaloplax* Carvalho & Reis, 2009: LBP 13108, 6, 36.4−37.8 mm SL, córrego sem nome, Saldanha Marinho, Rio Grande do Sul State.

*Hisonotus montanus* Carvalho & Reis, 2009: LBP 13051, 3, 26.4−27.2 mm SL, rio Goiabeiras, Vargem, Santa Catarina State; LBP 13055, 5, 24.8−31.9 mm SL, rio Canoas, Vargem, Santa Catarina State.

*Hisonotus nigricauda* (Boulenger, 1891): LBP579, 16, 34.1−40.1 mm SL, rio Guaíba, Eldorado do Sul, Rio Grande do Sul State.

*Hisonotus notatus* Eigenmann & Eigenmann, 1889: LBP 3472, 20, 21.0−34.3 mm SL, 3 c&s, 25.8−26.5 mm SL, rio Aduelas, Macaé, Rio de Janeiro State; LBP 10742, 25, 24.4−43.3 mm SL, rio Macabu, Conceição de Macabu, Rio de Janeiro State.

*Hisonotus notopagos* Carvalho & Reis, 2011: MZUSP 104943, 4, 35.3−37.3 mm SL, arroio Boici, Pinheiro Machado, Rio Grande do Sul State.

*Hisonotus oliveirai* Roxo, Zawadzki & Troy, 2014b: MZUSP 115061, 1, 26.4 mm SL, holotype, ribeirão Cambira, tributary of rio Ivaí, Cambira, Paraná State; LBP 13332, 1 23.2 mm SL, 1 c&s, 23.7 mm SL, paratype, rio Mourão, Campo Mourão, Paraná State; LBP 17578, 5, 25.4−30.4 mm SL, paratypes, rio Mourão, between Engenheiro Beltrão and Quinta do Sol, Paraná State; NUP 3578, 15, 24.7−28.1 mm SL, 2 c&s, 25.5−27.6 mm SL, paratypes, ribeirão Salto Grande, Maria Helena, Paraná State.

*Hisonotus paresi* Roxo, Zawadzki & Troy, 2014b: MZUSP 115062, 1, 26.2 mm SL, holotype, riacho Águas Claras, Santo Afonso, Mato Grosso State; LBP 13351, 9, 14.7−24.3 mm SL, paratype, riacho Águas Claras, Santo Afonso, Mato Grosso State; LBP 13352, 1, 23.7 mm SL, paratype, riacho Águas Claras, Santo Afonso, Mato Grosso State; NUP 10928, 2, 23.2−24.2 mm SL, paratype, 2 c&s, 23.6–24.2 mm SL, riacho Águas Claras, Santo Afonso, Mato Grosso State; NUP 10976, 3, 16.7−20.5 mm SL, paratype, riacho São Jorge, Santo Afonso, Mato Grosso State.

*Hisonotus piracanjuba* Martins & Langeani, 2012: LBP 17256, 9, 17.2−26.3 mm SL, 1 c&s, 27.1 mm SL, córrego sem nome, Morrinhos, Goiás State; NUP 5059, 1, 24.7 mm SL, córrego Posse, Anápolis, Goiás State; MZUSP 110491, 3, 17.5−24.4 mm SL, paratypes, rio Quente, Marcelãnia, Goiás State; NUP 10979, 3, 21.4−21.8 mm SL, ribeirão Bocaina, Piracanjuba, Goiás State.

*Hisonotus prata* Carvalho & Reis, 2011: MCP 40492, 18, 19.5−33.2 mm SL, rio da Prata, Nova Prata, Rio Grande do Sul State; LBP 9918, 14, 21.7−32.6 mm SL, Laguna dos Patos system, Nova Prata, Rio Grande do Sul State.

*Hisonotus ringueleti* Aquino, Schaefer & Miquelarena, 2001: FMNH 108806, 2, 25.7−32.2 mm SL, rio Quaraí basin, Uruguay; LBP 13148, 1, 24.5 mm SL, arroio Putiá, Uruguaiana, Rio Grande do Sul State.

*Hisonotus vireo* Carvalho & Reis, 2011: MZUSP 104946, 4, 30.4−39.5 mm SL, rio dos Sinos, Caraá, Rio Grande do Sul State.

*Hisonotus* sp. 1 n.: LBP 8276, 1 c&s, 25.6 mm SL, rio Verde Grande, Jaíba, Minas Gerais State.

*Hisonotus* sp. 2 n.: MZUSP 95687, 8, 19.8−21.5 mm SL, ribeirão da Anta, Gaúcha do Norte, Mata Grosso State.

*Hisonotus* sp. 3 n.: MZUSP 84157, 6, 20.1−23.6 mm SL, rio Manuel Alves, Porto Alegre do Tocantins, Tocantins State.

*Microlepidogaster arachas* Martins, Calegari & Langeani, 2013: LBP 10882, 3, 22.8−35.3 mm SL, rio Paraná basin, Arachas, Minas Gerais State.

*Microlepidogaster dimorpha* Martins & Langeani, 2011: LBP 10683, 2, 28.8−35.6 mm SL, rio Uberaba, Uberaba, Minas Gerais State.

*Otothyropsis marapoama* Ribeiro, Carvalho & Melo, 2005: LBP 4698, 6, 23.9−36.3 mm SL, ribeirão Cubatão, Marapoama, São Paulo State.

*Parotocinclus maculicauda* (Steindachner, 1877): LBP 2869, 15, 20.2−44.7 mm SL, rio Fau, Miracatu, São Paulo State.

*Parotocinclus prata* Ribeiro, Melo & Pereira, 2002: LIRP 1136, 38, 19.8−41.9 mm SL, paratypes, ribeirão Quiricó, Presidente Olegário, Minas Gerais State.

*Parotocinclus robustus* Lehmann & Reis, 2012: LBP 8258, 29, 18.7−39.1 mm SL, córrego Cachoeira, Bocaiúva, Minas Gerais State.

## Supplementary Material

XML Treatment for Hisonotus vespuccii
